# Intervention strategies for methicillin-resistant *Staphylococcus aureus* control in pig farming: a comprehensive review

**DOI:** 10.1186/s40813-025-00435-8

**Published:** 2025-04-03

**Authors:** Susanne Sawodny, Annemarie Käsbohrer, Laura Bröker, Clair Firth, Tatiana Marschik

**Affiliations:** 1 Centre for Food Science and Veterinary Public Health, Clinical Department for Farm Animals and Food System Science, University of Veterinary Medicine, Vienna, Austria; 2 Unit Epidemiology, Zoonoses and Antimicrobial Resistance, Department Biological Safety, German Federal Institute for Risk Assessment, Berlin, Germany

**Keywords:** Antimicrobial resistance (AMR), Biosecurity, Intervention measures, Methicillin-resistant *Staphylococcus aureus* (MRSA), Pig farming, Review

## Abstract

Methicillin-resistant *Staphylococcus aureus* (MRSA) poses a serious public health threat due to its zoonotic potential and resistance to several antibiotic classes. Pig farming is recognized as a key reservoir for livestock-associated MRSA, necessitating effective intervention strategies to mitigate its prevalence. The objective of this narrative review was to summarize the current knowledge on the approaches to control MRSA on pig farms. The review process involved a comprehensive search across three electronic databases focusing on studies from 2000 to 2024 in both English and German.

The review covers intervention measures including reduced antimicrobial use, cleaning and disinfection, air filtration, and bacteriophage application. Key findings indicate that, while interventions such as cleaning and disinfection and air filtration, can effectively reduce environmental MRSA loads, these measures are often insufficient for long-term control due to frequent recontamination, especially restocking with MRSA-positive animals. Eradication was shown to be effective in low-prevalence regions such as Norway, however, logistical and ethical challenges limit its feasibility in areas with high MRSA prevalence. Additional interventions, such as reduced antimicrobial use and sow washing, provided inconsistent results.

Overall, the findings highlight the need for a multifaceted approach, combining several interventions tailored to regional MRSA prevalence, farm management practices, and available resources. Such an integrated strategy is essential for sustainable MRSA control in pig farming, thereby supporting the global One Health initiative aimed at mitigating antimicrobial resistance.

## Background

The global increase in antimicrobial-resistant bacteria presents a major public health challenge. Nearly 5 million deaths annually are attributed to limited treatment options due to antimicrobial resistance (AMR), and without effective interventions, this number is expected to reach 10 million per year by 2050 [1, 2]. AMR imposes considerable economic burdens, with costs estimated to exceed €100 trillion by the same year [1, 3, 4]. Often referred to as the ‘silent pandemic’, AMR is an invisible crisis affecting humans, animals, and the environment. This hidden emergency has prompted various political initiatives over the past decades aimed at ensuring the future effectiveness of antimicrobial agents [2, 5].

In 2015, the World Health Organization (WHO) responded by adopting the Global Action Plan to tackle the growing threat of AMR worldwide. This plan emphasizes several key objectives, including raising awareness, strengthening knowledge, reducing infection rates through prevention and biosecurity measures, and promoting the more targeted use of antimicrobial agents [6]. Closely aligned with this effort, the 2024 WHO Bacterial Priority Pathogens List identifies bacterial pathogens of global concern due to their AMR [1, 7]. Methicillin-resistant *Staphylococcus aureus* (MRSA), which is known to have developed resistance to beta-lactam antibiotics and, in some cases, other antibiotic classes, is included on this list as a high-priority pathogen, driving research, improved diagnostics, and strengthened infection control practices to mitigate its global impact [7, 8].

MRSA is generally classified into three categories: Hospital-associated MRSA (HA-MRSA), community-associated (CA-MRSA), and livestock-associated (LA-MRSA). Although these classifications are based on epidemiological distinctions, overlaps between them do occur. HA-MRSA and CA-MRSA typically affect humans, while LA-MRSA predominantly affects animals [9]. Lineage CC398 LA-MRSA is the most common clonal complex identified in pigs and is usually resistant to tetracyclines in addition to beta-lactam antibiotics [10]. In both animals and humans, MRSA can exist as a commensal organism or act as an opportunistic pathogen, colonizing nasal passages and skin. Besides asymptomatic carriages, it can cause a range of infections, including skin, wound, soft tissue, and septicemia [11, 12]. Various transmission routes have been documented, with both direct and indirect spread occurring between and within species, including zoonotic transmission. Zoonotic transmission of LA-MRSA is common, especially in individuals who frequently interact with colonized animals, such as veterinarians, farmers, and slaughterhouse workers [12–15].

There is strong scientific evidence that the high prevalence of LA-MRSA in pig farms, combined with the primarily asymptomatic nature of the infection in pigs, indicates that pigs are an important reservoir for MRSA [15–17]. Several factors contribute to the prevalence of MRSA in pigs. Key factors include herd size and stocking density, as direct contact between animals facilitates transmission. Additionally, the age of the pigs and the type of production system play critical roles, influencing factors such as immune system responses, animal density, and levels of antibiotic usage [18, 19].

According to global data, the prevalence of LA-MRSA varies between regions worldwide, as seen in reports from Asia and the European Union (EU) [9, 17, 20–22]. In Asia, prevalence in pigs, identified as the most frequently affected MRSA-positive animals, varies across countries, from 0.9% in Japan to 42% in Taiwan. These differences are likely influenced by a variety of factors such as antimicrobial use, housing systems, sampling locations, and pig movement [20, 22]. A similar variation can be observed in the EU, where some Member States (e.g., Denmark and the Netherlands) [23, 24] have implemented voluntary monitoring programs. Recent prevalence data is included in the EU Summary Report on Antimicrobial Resistance in Zoonotic and Indicator Bacteria from Humans, Animals and Food [9, 21], according to which pigs have been consistently identified as the most frequently affected MRSA-positive animals in the EU. Prevalence rates vary widely, ranging from 80% positive fattening pigs in Belgium to no detection in Norway. Prevalence in breeding pig herds is slightly lower, from 45% MRSA-positive animals in Belgium to zero detected cases in Norway. These findings are consistent with the 2008 Baseline Survey on the Prevalence of MRSA in EU Breeding Pig Holdings, where the prevalence ranged from 0% in Norway, 5% in Austria, to 40% in Belgium and 43% in Germany. Both Belgium and Germany, therefore, exceeded the regional EU average in that study of 23% [25].

Biosecurity measures play a critical role in combating the spread of livestock pathogens, particularly in pig farms. These practices are designed to prevent the introduction and transmission of pathogens through a combination of hygiene protocols, control of animal movement, and environmental management [26–29]. In the EU, general biosecurity requirements are defined and regulated under the *Animal Health Law* (AHL) (Regulation (EU) 2016/429), with additional recommendations provided by the European Food Safety Authority (EFSA) [30–32]. Targeted efforts to prevent LA-MRSA must align with general biosecurity standards, as their adherence directly impacts the effectiveness of MRSA control measures.

A number of recent studies have focused on evaluation of measures and interventions to reduce or prevent MRSA occurrence in livestock farming globally. However, to the authors’ knowledge, no comprehensive review has yet summarized the scientific evidence on the effectiveness of measures to reduce or eliminate MRSA in pig farms. Identifying the most successful interventions in different contexts is essential for developing tailored strategies that protect public health and support the global One Health initiative.

## Materials and methods

This narrative review was conducted in accordance with PRISMA guidelines. To systematically identify and compare all relevant articles, a structured literature search was performed using three scientific databases: PubMed, Clarivate (Web of Science), and Scopus. The search focused on three critical aspects: (1) MRSA, (2) intervention measures and their effectiveness, and (3) the pig as the target animal species. Search terms from each category were combined with Boolean operators (AND/OR/NOT) and applied across all three databases. The screening included all articles published between January 2000 and March 2024 in both English and German.

For the English search, following terms were used:

(“MRSA” OR “Staphylococcus aureus” OR “LA-MRSA”) AND (“measur*” OR “prevent*” OR “program*” OR “eradication” OR “strateg*” OR “effectiv*” OR “reduction” OR “elimination” OR “disinfect*”) AND (“pig” OR “pigs” OR “swine” OR “sow”) NOT “guinea pig”.

For the German search, a corresponding combination was applied.

The initial screening of extracted articles was based on their titles, with exclusion criteria applied if the title did not indicate a focus on measures for reducing MRSA or was not related to pigs. Next, abstracts were reviewed based on the following questions, with the full manuscript consulted if clarification was needed. To meet the inclusion criteria, questions 1 and 2, as well as at least one sub-item from question 3, had to be answered affirmatively:

1. Is the focus on pig herds?
2. Is MRSA investigated?
3. Are potential measures considered regarding:
  - Preventing the entry of MRSA into a farm.
  - Preventing the spread and multiplication of MRSA within a farm.
  - Preventing emissions and thus further spread to other farms.
  - Reducing the prevalence of MRSA on a farm.
4. Is the effectiveness of the measures evaluated?

The categorization of question 3 into subcategories was based on the European Medicines Agency (EMA) and EFSA Joint Scientific Opinion on Measures to Reduce the Need to Use Antimicrobial Agents in Animal Husbandry in the European Union, and the Resulting Impacts on Food Safety (RONAFA) [33]. This scientific opinion, reflected in our selection criteria, divides disease prevention measures into three categories: primary prevention, which includes measures to prevent the introduction of pathogens into farms and their spread between farms; secondary prevention, which focuses on reducing transmission and spread within a farm; and tertiary prevention, which involves measures aimed at enhancing animals’ ability to cope with pathogens. It should be noted that tertiary prevention was not addressed in this review, as the focus was primarily on interventions aimed at preventing the introduction and spread of MRSA within and between pig farms. For question 4, which focuses on evaluating the effectiveness of the intervention measures, the assessment was based on the outcomes reported in each study concerning MRSA prevalence. Each study was reviewed for its ability to demonstrate a reduction in MRSA occurrence, with a particular focus on quantitative data.

## Results

**Fig. 1:**
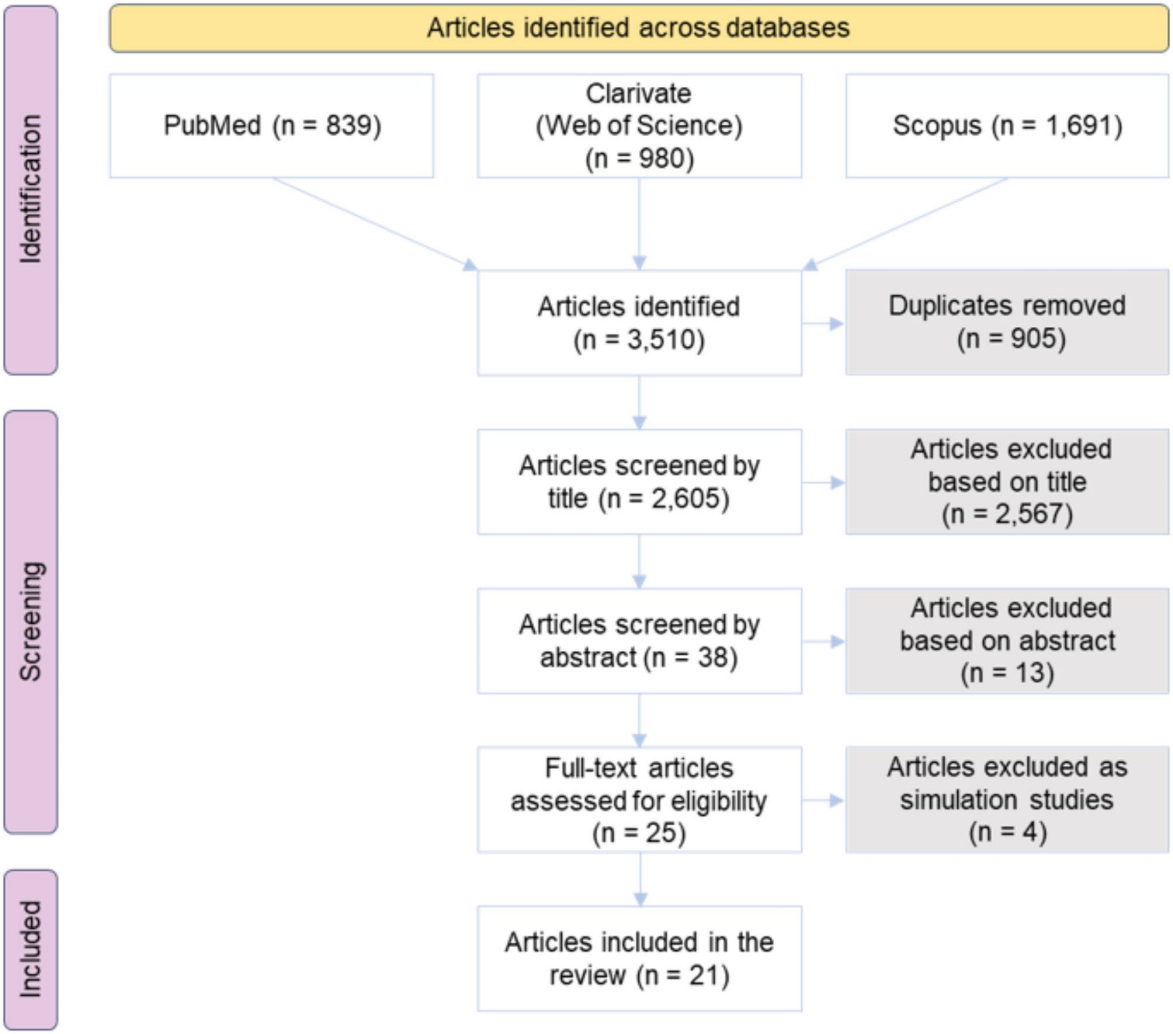
Review process flow chart

Figure 1 illustrates the search strategy and results. A total of 3,510 articles were initially identified across the selected databases. After removing 905 duplicates, 2,605 unique articles remained for title screening. Of these, 38 articles were selected for further screening based on their abstracts. Following further evaluation, 25 full-text articles were assessed for eligibility. Four studies were excluded as they were classified as simulation studies. In the end, 21 articles met the inclusion criteria and were included in the final review.

The annual number of published articles showed an upward trend from 2000 to 2024, with more than 75% of the studies (16/21) published between 2014 and 2024. The included studies covered 8 different European countries, Australia and the United States, demonstrating geographic variability. The highest concentration of studies was observed in Germany (*n* = 5), Belgium (*n* = 4), and the Netherlands (*n* = 3).

The reviewed articles exhibited diversity in study designs and provided evidence of the effectiveness of the implemented measures in reducing MRSA prevalence.

**Fig. 2:**
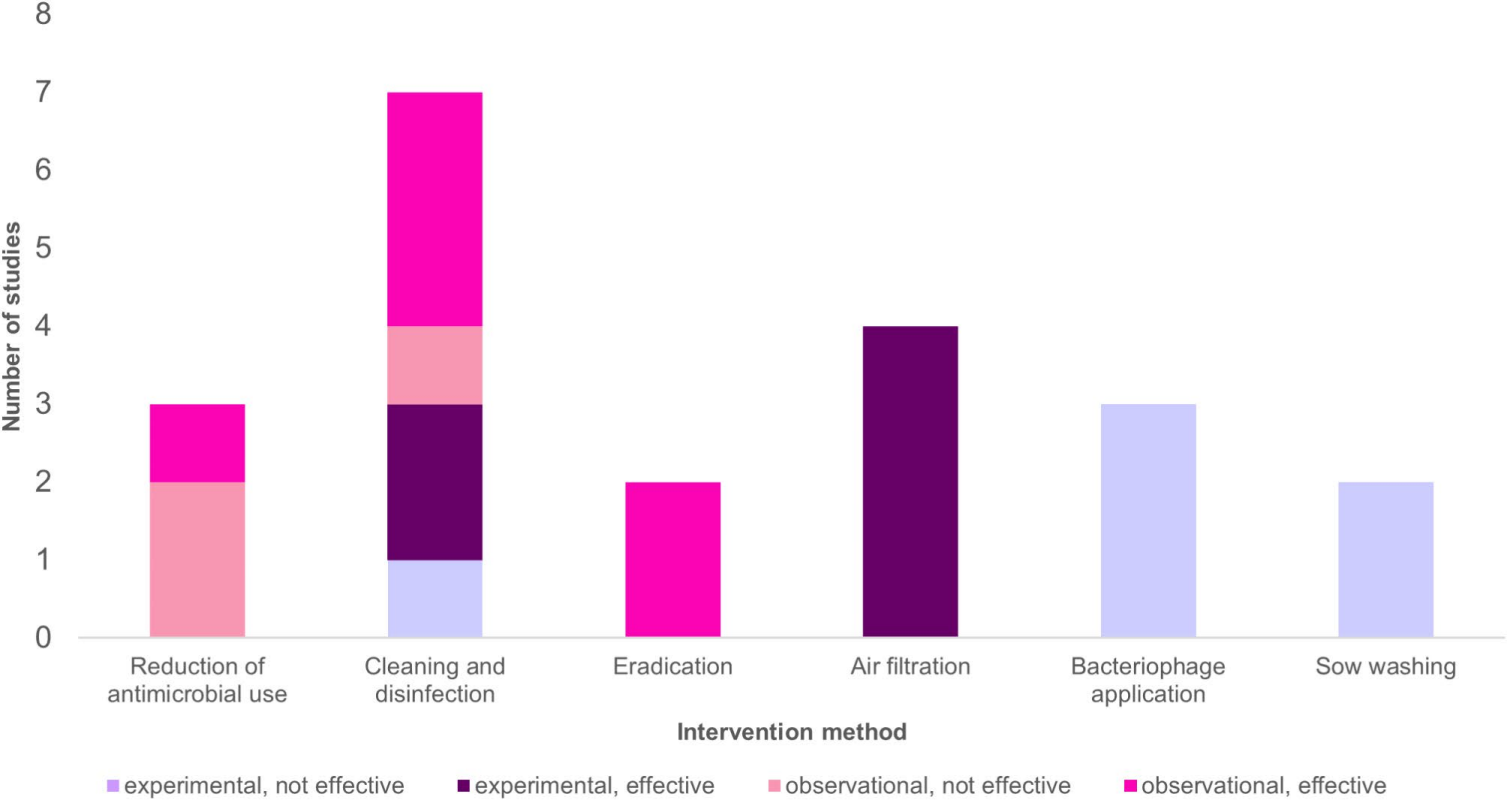
Stacked bar chart illustrating the number of studies by intervention measure, study design, and effectivity

Moreover, the studies were grouped according to the most commonly implemented intervention strategies against MRSA, which included: reducing antimicrobial use, cleaning and disinfection (C&D), eradication, air filtration, bacteriophage application, and sow washing. Table 1; Fig. 2 provide a comprehensive overview of these interventions, their applications, and their effectiveness across the reviewed studies.

**Table 1:** Overview of the publications included in the review

Classification*/ Prevention strategy	Intervention method	Study design	Sampling details	Effectivity	Country	Author, Year	Reference
**Preventing the entry of MRSA into a farm**							
	Reduction of antimicrobial use	Observational	Animals (abattoirs, *n* = 7)	No	Netherlands	Dierikx et al. (2016)	[35]
		Observational	Humans, animals (pig farms, *n* = 36)	Yes	Netherlands	Dorado-García et al. (2015)	[39]
		Observational	Animals (pig farms, *n* = 2)	No	Portugal	Lopes et al. (2019)	[40]
**Preventing the spread and multiplication of MRSA within** ** a farm**							
	Cleaning and disinfection	Experimental	Environment (pig farms, *n* = 1)	Yes	Belgium	Luyckx et al. (a) (2016)	[49]
		Experimental	Environment (pig farms, *n* = 1)	No	Belgium	Luyckx et al. (b) (2016)	[50]
		Experimental	Environment, animals (pig farms, *n* = 1)	Yes	Germany	Kobusch et al. (2020)	[42]
		Observational	Environment, humans, animals (pig farms, *n* = 1)	Yes	Germany	Schmithausen et al. (2015)	[43]
		Observational	Environment, animals (pig farms, *n* = 1)	Yes	Germany	Schollenbruch et al. (2021)	[27]
		Observational	Environment (pig farms, *n* = 6)	Yes	Italy	Merialdi et al. (2013)	[26]
		Observational	Environment (pig farms, *n* = 20)	No	Italy	Scollo et al. (2023)	[46]
	Eradication	Observational	Environment, humans, animals (pig farms, *n* = 9)	Yes	Norway	Elstrøm et al. (2019)	[52]
		Observational	Environment, animals (pig farms, *n* = 2)	Yes	Norway	Karlsen et al. (2021)	[53]
**Preventing emissions and thus further spread to other farms**							
	Air filtration	Experimental	Air (in vitro, in *n* = 1)	Yes	Australia	Tenzin et al. (2019)	[58]
		Experimental	Air (pig farms, *n* = 2)	Yes	Germany	Clauss et al. (2013)	[54]
		Experimental	Air (pig farms, *n* = 1)	Yes	Germany	Schulz et al. (2013)	[56]
		Experimental	Air (pig farms, *n* = 1)	Yes	USA	Ferguson et al. (2015)	[55]
**Reducing the prevalence on a farm**							
	Bacteriophage application	Experimental	Environment, animals (pig farms, *n* = 1)	No	Finland	Tuomala et al. (2021)	[62]
		Experimental	Animals (in vitro, in vivo (pig farms, *n* = 1), ex vitro)	No	Netherlands	Verstappen et al. (2016)	[63]
		Experimental	Environment, animals (pig farms, *n* = 1)	No	Switzerland	Honegger et al. (2020)	[64]
	Sow washing	Experimental	Environment, animals (pig farms, *n* = 2)	No	Belgium	Pletinckx et al. (2013)	[66]
		Experimental	Animals (pig farms, *n* = 4)	No	Belgium	Verhegghe et al. (2013)	[67]

*The publications are categorized by prevention strategy and intervention method including details on the study design, sampling, and effectiveness outcomes. MRSA: Methicillin-resistant *Staphylococcus aureus*

In the following section, we briefly describe the reviewed studies, categorized according to the most commonly implemented intervention measures as listed in Table 1; Fig. 2.

### Reduction of antimicrobial use

Due to the relatively high use of antibiotics in the Dutch veterinary sector in the early 2000s [34], the national government introduced strict limits on their use in livestock in 2010. As a result, antibiotic consumption decreased by approximately 50% in the following years, prompting extensive research on antibiotic reduction measures in pig farming [35, 36].

A study conducted by Dierikx et al. [35] aimed to evaluate whether the 50% reduction in the use of antimicrobials led to a lower prevalence of MRSA in slaughter pigs compared to findings from a 2005 study. There, Neeling et al. [37] reported an overall MRSA prevalence of 39% and a batch prevalence of 81% in Dutch abattoirs. Ten years later, Dierikx et al. [35] examined 10 fattening pigs again from each of the 56 sampled slaughter batches, sourced from seven different abattoirs, using nasal swabs. The batch prevalence was 100%, indicating that at least one pig in each batch tested positive for MRSA, with an overall prevalence of 83%. It was concluded that the reduced antimicrobial use at national level had not yet had an effect on MRSA prevalence in pigs entering the abbatoir. Dierikx et al. [35] noted that cross-contamination between pigs during transportation or while the pigs were in the lairages cannot be ruled out, as confirmed by previous research [38], which suggests that MRSA prevalence at abattoirs is often higher than on pig farms and may have obscured the true impact of the reduction in antibiotic use between 2005 and 2015 on MRSA prevalence.

Dorado-García et al. [39] conducted an 18-month study involving 36 pig farms in the Netherlands, with sampling carried out at 6-month intervals. Tailored interventions were designed individually for each farm, focusing on reducing antibiotic use, improving hygiene practices, and altering animal contact patterns. During the study period, the defined daily dosages per animal per year for antimicrobial use on the farms decreased by an average of 44%. Consequently, the number of MRSA-positive farms showed a slight decline, dropping from 31 to 29. Farms with higher antimicrobial use had a substantial greater number of MRSA-positive pigs. The authors concluded that reducing antimicrobial use could potentially lower MRSA prevalence in pigs.

A study by Lopes et al. [40] investigated the impact of banning certain antimicrobial substances on MRSA prevalence and AMR on two Portuguese pig farms. The focus was specifically on reducing antimicrobials used as feed additives for prophylactic purposes. Given the high prevalence of LA-MRSA (99%) observed in 2016 on pig farms using such additives [41], a complete ban on these substances - amoxicillin, colistin, and zinc oxide - or at least a restriction on colistin use was implemented. However, the MRSA prevalence in both farms remained largely unchanged in 2018, at 96%. The authors concluded that while the ban on antimicrobial substances as prophylactic feed additives increased the sensitivity of MRSA to several antimicrobials, it did not lead to a reduction in its overall prevalence.

### Cleaning and disinfection

The reviewed studies demonstrated a range of C&D protocols and their effectiveness in controlling MRSA prevalence in pig farming. Most interventions led to a reduction in contamination, with outcomes varying based on the specific measures applied. Details on the C&D protocols, sampling methods, and results are presented in the following section and summarized in Table 2.

**Table 2:** Overview of the studies focusing on cleaning and disinfection (C&D) measures

Investigated pig group	C&D process	Cleaning agent used	Disinfectant used	Effectiveness outcome	Author, year	Reference
Six nursery pig units (two groups: Control group with C&D and a test group with CE)	Control group: Manual removal with cold water, 24- hour empty period, soaking with sodium hydroxide, high-pressure cleaning, disinfection. Test group: Manual removal with cold water, 24-hour empty period, soaking with 1.5% Probiotics cleaner, rinsing with warm water	Control group: Sodium hydroxide Test group: Bacillus spp. Spores and enzymes	Control group: Glutaraldehyde and quaternary ammonium compounds Test group: No disinfection	Control group: MRSA prevalence decreased to 4% immediately after C&D Test group: MRSA prevalence only dropped to 20% immediately after CE Prevalence increased in both groups in the following weeks up to the levels before C&D and CE	Luyckx et al. (a) (2016)	[49]
Six nursery pig units	Soaking with water, cleaning with hot water (80 °C) and disinfection with glutaraldehyde/quaternary ammonium compounds. A 10-day vacancy period after disinfection with monitoring of bacterial loads	No specific cleaning agent used	Glutaraldehyde and quaternary ammonium compounds	No significant impact during the 10-day vacancy period; minimal changes observed, indicating limited efficacy of prolonged vacancy periods .	Luyckx et al. (b) (2016)	[50]
Piglet-rearing compartments during weaning phase	Standard C&D process: manual removal of manure, soaking with water, high-pressure cleaning, foam cleaner, drying, and disinfection	Sodium hydroxide-based foam cleaner	Hydrogen peroxide and peracetic acid	LA-MRSA prevalence reduced from 71% to 2–3% after C&D, indicating high effectiveness under described conditions	Kobusch et al. (2023)	[42]
Model pig farm (old and new stables)	High-pressure cleaning, foam cleaning, surface disinfection, hot nebulization, and strict hygiene protocols	Potassium hydroxide solution, amphoteric surfactants	Glutaraldehyde, formaldehyde, benzylalkyl dimethyl ammonium chloride	MRSA eradicated initially, but it reappeared within 2 days of restocking, indicating high initial efficacy but recontamination from external sources	Schmithausen et al. (2015)	[43]
Fattening pigs on straw bedding (two groups: (A) straw bedding plus C&D vs. (B) straw bedding plus simple cleaning)	Group A: Manual removal of manure, soaking with water, high-pressure cleaner, sodium hydroxide foam cleaner, drying, foam disinfectant. Group B: Same as Group A besides application of foam cleaner was skipped, and no disinfection was applied	Sodium hydroxide foam cleaner	Group A: Formic acid	LA-MRSA prevalence reduced to 28% in Group A and to 0% in Group B after 16 weeks, indicating higher effectiveness of cleaning only in reducing LA-MRSA colonization	Schollenbruch et al. (2015)	[27]
Six farrow-to-finish pig herds	High-pressure cleaning (hot and cold water, depending on herd) with application of glutaric aldehyde, quaternary ammonium compounds, or alkyl amines disinfectants	Detergent used before disinfection in all herds	Glutaric aldehyde, quaternary ammonium compounds, alkyl amines, inorganic peroxygen, organic acids	MRSA contamination reduced from 50–19% after C&D, with the greatest reduction observed in farrowing crates. C&D was not effective in eliminating MRSA completely from the environment	Merialdi et al. (2013)	[26]
20 pig farms (fattening and breeding/nursery)	Manual removal of organic material, soaking with warm water, detergent application, surface disinfection, and fogging method. Regular monitoring of ATP values for hygiene status	No specific cleaning agent used	No specific disinfectant used	LA-MRSA prevalence was not significantly impacted	Scollo et al. (2023)	[46]

CE: Competitive exclusion; C&D: Cleaning and disinfection; LA-MRSA: Livestock-associated Methicillin-resistant *Staphylococcus aureus*; MRSA: Methicillin-resistant *Staphylococcus aureus*

Kobusch et al. [42] conducted a single-blind study to assess the efficacy of routine C&D procedures in decontaminating LA-MRSA in pig farms in Germany. First, environmental samples were collected in an empty barn both before and after the C&D procedure. Once the pigs were housed, sampling was conducted three times throughout the rearing phase, targeting both the barn environment and the newly housed rearing pigs. The C&D protocol involved several steps: manual manure removal, a soaking phase lasting several hours, high-pressure cleaning followed by foam cleaning with sodium hydroxide, and a second high-pressure cleaning. The barn was then left to dry for 18 h before being subjected to foam disinfection using hydrogen peroxide and peracetic acid.

The results showed that, on average, prior to C&D, 72% of environmental samples were LA-MRSA-positive, with an increased prevalence of positive samples (80%) in areas the animals had access to. Interestingly, the prevalence of positive samples was slightly higher for the easy-to-clean areas (73.9%) compared to difficult-to-clean areas (70%). Following C&D the prevalence of positive environmental samples dropped significantly to 2.7%. On the day of housing, 71.7% of piglets and 1.7% of surfaces tested positive for LA-MRSA. However, after seven weeks, 100% of the piglets and 83.7% of the surfaces tested positive. These findings suggest that while C&D can significantly reduce LA-MRSA in the environment, housing LA-MRSA-positive pigs leads to rapid recontamination. The authors concluded that in well-managed livestock farms, decontamination of a LA-MRSA positive barn is possible. However, for effective eradication, restocking should only be done with LA-MRSA-negative pigs and other potential sources of contamination, such as LA-MRSA-positive humans, should be addressed through decolonization measures.

Schmithausen et al. [43] conducted a study evaluating the measures taken to eliminate MRSA and *Enterobacteriaceae* expressing extended-spectrum beta-lactamase (ESBL-E). A decontamination process was carried out on a farm that tested positive for these pathogens. Firstly, the pigs were removed from the farm and culled. Subsequently, a decontamination procedure was conducted in the barn, and additionally a new barn was built. The process involved dismantling most of the interior, followed by high-pressure cleaning, foaming and purifying the barn with potassium hydroxide solution and disinfection using glutaraldehyde, formaldehyde, and products containing ammonium chloride. Environmental and nasal samples from pigs were collected before and after decontamination process over the course of one year. All environmental samples were negative after the decontamination process, indicating its effectiveness. However, after pig production resumed, MRSA prevalence increased, and after one year, MRSA colonization in pigs was 31.6%, though it was a different spa-type than before the decontamination. The authors suspect a new entry through purchased pigs, suggesting that intensive screening for MRSA in purchased animals is necessary to prevent reintroduction of the pathogen.

Schollenbruch et al. [27] investigated whether different cleaning methods in straw bedding husbandry impact LA-MRSA prevalence in fattening pigs. The study divided pigs, all of which were LA-MRSA-positive, into two groups. The barn for Group A underwent C&D before housing, involving removal of straw and manure, a soaking phase, high-pressure washing, foam cleaning with sodium hydroxide, a second high-pressure washing, an 18-hour drying phase, and foam disinfection. The barn for Group B was only cleaned through removal of straw and manure, followed by a soaking phase and high-pressure washing without a foam cleaner. During the consecutive fattening period, environmental and nasal samples were collected from the pigs at five different time points.

After C&D of the barn and before the animals were housed, all environmental samples were MRSA-negative. At housing, 100% of the pigs were nasally MRSA colonized. The prevalence in the environment increased and after one week it reached 90% in Group A and 100% in Group B. In the further course of the fattening period, the MRSA prevalence decreased both in the pigs and in the environment, whereby the prevalence in Group B was lower than in Group A. At the end of the fattening period, all pigs in Group B were MRSA-negative and 89% of the environmental samples were negative. In contrast, only 72% of pigs and 67% of environmental samples were negative in Group A. The study concluded that cleaning alone might be more effective than C&D in reducing LA-MRSA prevalence in pigs. Since both groups were housed on straw, the impact of this housing condition on prevalence remains inconclusive. The authors suggest that the use of straw may be an important factor to consider, as other studies have indicated that alternative farming methods may have a positive impact on reducing LA-MRSA prevalence [44, 45].

A study from Italy by Merialdi et al. [26] analyzed the differences in MRSA prevalence in the various production phases (farrowing, weaning, growing, and fattening phase), and the impact of C&D on the environmental MRSA prevalence. Dust samples were collected from different units of six farrow-to-finish farms both before and after C&D. As all farms had different management systems, the C&D protocols varied between farms. Overall, the prevalence decreased from an average of 50–19%, although there were large differences between production units in terms of MRSA prevalence and reduction. Before C&D, the lowest percentage of MRSA-positive samples was observed in the fattening units (31.7%) compared to 60% in the growing pens. The greatest reduction in MRSA prevalence after C&D was found in the farrowing units, where it decreased from 53.3 to 1.7%, while the prevalence in the growing pens decreased to 35%. This significant difference was attributed to the use of easy-to-clean materials in the crate stalls of the farrowing units. The authors overall concluded that C&D measures can reduce, but not completely eliminate, MRSA from the animals’ environment.

Scollo et al. [46] investigated the effectiveness of improving biosecurity measures, particularly C&D, on 20 pig farms using tailored plans to reduce MRSA prevalence over a 12-month period. The authors used a questionnaire and a farm visit to assess the biosecurity status of each farm and subsequently developed a customized biosecurity improvement plan. Measures included enhancing hygiene sluices for employees and visitors, improving C&D procedures, and implementing a rodent control plan. Over the 12 months, the farms adopted these enhanced biosecurity measures and participated in theoretical and practical hygiene management training, which included a C&D protocol training in an empty barn (removal of organic material, pre-soaking, wet cleaning, rinsing, drying, disinfection, and a 7-day empty period). At the beginning of the study, prior to implementing the measures, C&D in empty barns did not lead to a significant reduction in MRSA prevalence. After 12 months, samples were again collected before and after a standardized C&D protocol. No significant decrease in MRSA prevalence was observed either before and after C&D or across different sampling times. In light of the high MRSA prevalence determined, the authors stressed the need to improve biosecurity and farm hygiene.

As MRSA is becoming increasingly resistant to conventional disinfectants, alternative cleaning methods must be considered [47, 48]. One such alternative is competitive exclusion (CE), which was investigated by Luyckx et al. (a) [49] with respect to MRSA in pig barns. In their study, six nursery units within a pig farm were divided into a control group and a test group. The control units underwent a standard C&D protocol after piglets were removed from the barn, while the test units followed a CE cleaning protocol. In both groups, manure was initially removed with cold water followed by a 24-hour empty period. In the control group, the next step of the C&D protocol involved soaking with 2% sodium hydroxide and rinsing with cold high-pressure water, followed by disinfection with 1% glutaraldehyde and quaternary ammonium compounds, and a subsequent 2-week empty period. In the test group, the CE protocol was carried out, which included the following steps: the units were soaked for 10 min with a solution of 1.5% probiotic bacteria consisting of *Bacillus* spp. spores of five different species in a concentration of 8.5 and 7.5 log colony forming units (CFU)/mL, in 40 °C warm water and then rinsed with warm water. During the subsequent 2-week empty period and the production phase, the units in the test group were sprayed with probiotic bacteria 2 to 3 times per week.

Swab samples were collected before cleaning, immediately afterwards, and in the first and fifth weeks after piglets were housed. No difference in MRSA prevalence was observed between the groups prior to cleaning, with a prevalence of around 80%. After cleaning, MRSA prevalence in the control group decreased to 4%, while in the test group, it only dropped to 20%. Sampling in weeks 1 and 5 after cleaning revealed MRSA prevalence levels similar to those observed before cleaning. In conclusion, the CE protocol was not as effective in reducing MRSA prevalence as the standard C&D protocol and the authors concluded that the CE protocol does not appear to be a valuable alternative for MRSA control in pig barns.

In a study conducted by Luyckx et al. (b) [50], the effect of a C&D protocol in combination with a prolonged 10-day vacancy period on bacterial load, including MRSA detection, was investigated in pig nursery units. The experiment was repeated three times. After the pigs were removed, the housing units were soaked in water, followed by rinsing with hot water and disinfection using a solution of glutaraldehyde and quaternary ammonium compounds. Subsequently, the units remained unoccupied for 10 days. Environmental samples were collected at various time points: before C&D, and on days 1, 4, 7, and 10 after C&D.

The initial MRSA prevalence in environmental samples was 16% before C&D. Following C&D, MRSA prevalence dropped to 7% but then increased to 14% by day 4 of the vacancy period before decreasing again to 8% on day 10. Based on these results, the authors concluded that an extended vacancy period does not significantly reduce MRSA load in the environment. This finding raised the question of whether prolonged vacancy periods provide any real benefit or simply lead to financial losses due to fewer production cycles [50].

### Eradication

Since 2014, a comprehensive surveillance program aimed at eradicating MRSA from the pig population through targeted slaughter and culling measures has been implemented in Norway [51]. This program has successfully kept MRSA prevalence at a low level, and there have been no significant outbreaks in the last decade [9, 21]. Two Norwegian studies have examined the effectiveness of these measures on MRSA-positive animals, providing valuable insights into the success of the eradication strategy.

A study by Elstrøm et al. [52] described an outbreak in 2015 in which nine farms tested positive for MRSA. The outbreak was traced back to a farm that had spread MRSA to the other farms through the sale of positive pigs. The affected farms underwent decontamination, and depopulation measures were implemented. Once MRSA was no longer detected, the farms were repopulated with MRSA-negative pigs. Subsequent sampling confirmed the absence of MRSA in all repopulated farms over a period of around 1.5 years.

A case report published in 2021 by Karlsen et al. [53] described the outcomes of MRSA eradication measures on two affected pig farms. The farms were connected, as Farm B, a fattening farm, had purchased pigs from Farm A, a breeding farm, leading to its inclusion in contact tracing and subsequent testing. Individual eradication protocols were developed for each farm, with Farm A undergoing more extensive measures. On Farm A, the protocol included removing all interior and equipment, disposing of them, and renovating all interior surfaces of the barn. Additionally, the oldest barn was completely demolished. In contrast, Farm B did not make any changes to the interior structure and only carried out C&D. Both protocols involved depopulating the pig herds, and all environmental samples were required to test negative before repopulation. The eradication programs were successful on both farms, demonstrating that both extensive and less extensive protocols can be effective in eliminating MRSA. However, it should be noted that Farm B, being a fattening farm with a simpler operational model and fewer facilities than Farm A, likely benefits from a structure that allows standard C&D protocols to be more effective.

### Air filtration

Since *Staphylococcus aureus* (*S. aureus*) can be transmitted via aerosols, there is a risk of MRSA spreading through the air from one farm to another. The significance of this transmission route is well-documented [54, 55]. To mitigate this spread, several research groups have focused on cleaning exhaust air from pig barns, with their findings described in the following chapter.

A German study by Clauss et al. [54] evaluated the effectiveness of two different air filtration systems in reducing LA-MRSA in the exhaust air of two pig fattening farms. The two systems, i.e., trickle bed reactor and a three-stage system with different filters, which were already in place in the farms, were sampled ten times over a five-month period at intervals of every 2–3 weeks. Both systems achieved an average reduction rate of over 90% in LA-MRSA concentrations between the emitted air (before the filter) and the purified air (after the filter). However, significant fluctuations were observed, with minimal differences in LA-MRSA levels before and after filtration on certain days. The authors attributed these variations to differing climatic conditions, process parameters such as ventilation rates on sampling days, and potential statistical distortions, as minor fluctuations can have a large impact when LA-MRSA concentrations in emitted air are low. Despite not fully eliminating LA-MRSA, the filtration systems significantly reduced its presence, thereby minimizing the bacterium’s release into the environment.

A German study by Schulz et al. [56] evaluated the efficiency of an air purification system combined with UV irradiation. The system was installed on a fattening pig farm, where MRSA, along with other bacteria and fungi, were assessed. Sampling was conducted on four different days over a three-week period. Both the air filter and UV irradiation independently reduced microbial concentrations, but their combination was the most effective, achieving a significant MRSA reduction of over 99%. While the system appears capable of reducing airborne MRSA, the authors questioned its feasibility due to the high water consumption and numerous devices required in a barn, which could result in substantial costs.

The study by Ferguson et al. [55] evaluated the effect of two biofiltration systems - hardwood chips, and western red cedar shredded bark - in eliminating MRSA on pig farms. The filters were connected to an existing exhaust airduct, and aerosol and airborne dust samples were collected in a pig nursery over four days. Results showed that hardwood chips reduced MRSA particulate matter with a mean particle size of 5.85 μm by 92%, while western red cedar achieved a 100% reduction (however, this effectivity decreased for smaller particle sizes). The authors concluded that biofilters are a viable method for reducing MRSA emissions from pig farms.

An alternative to conventional exhaust air filtration is air filtration with an electrochemically activated solution (ECAS). ECAS has already proven effective in disinfection in medicine and in food technology [57]. The study by Tenzin et al. [58] dealt with the effectiveness of ECAS4^®^ (ECAS4 Australia Pty Ltd, Australia) fogging for the decontamination of a pig farm between herds. The experiment was conducted in two steps. In the in vitro trial, low concentrations of ECAS4 in water demonstrated effective killing of MRSA isolates. In the in vivo trial, an empty weaning room was fogged with ECAS4 for 3 min every 30 min over a 5-hour period. Air samples were then collected and showed a 99.99% reduction in total bacterial count after fogging with ECAS4 anolyte. Since MRSA was not specifically detected in the in vivo trial, but efficacy was shown in the in vitro trial, a potential next step would be to repeat a similar test with specific MRSA detection in vivo.

### Bacteriophage application

Phage therapy is increasingly considered to be an alternative to the conventional use of antibiotics. Due to their high specificity and ability to target different bacterial receptors, phages may offer a promising approach to combating bacterial infections. The development of phage cocktails, which consist of multiple phages targeting various bacterial strains, is seen as a particularly effective strategy to reduce antibiotic usage [59]. As several studies have already demonstrated the positive effects of bacteriophage use in treating various diseases in both humans and animals [60, 61], veterinary research is increasingly focusing on the use of phages as a potential treatment option for MRSA in pig farms.

Tuomala et al. [62] conducted a study to evaluate the efficacy of phage treatment in eradicating LA-MRSA in healthy carrier pigs. Nineteen MRSA-positive weaning piglets were divided into a test group and a control group. A phage cocktail was applied to the skin and nostrils of the test group three times over six days. During the experiment, nasal and skin swabs were taken to analyze the bacterial and phage counts in the nostrils and on the skin. Blood samples were also collected to detect the formation of antiphage antibodies, and environmental samples were analyzed as well.

None of the pigs were completely negative for MRSA during the entire follow-up period. Half of the pigs remained MRSA-positive throughout the study, while the others tested negative at least once. Phages were detected in both MRSA-positive and MRSA-negative pigs, indicating that the presence of phages does not necessarily correlate with MRSA eradication. No antiphage antibodies were found in the blood samples, suggesting that resistance or neutralizing antibodies were not responsible for the limited effectiveness of the treatment. Consistent with other studies, Tuomala et al. [62] concluded that phage treatment did not lead to a significant reduction in MRSA colonization in pigs.

Verstappen et al. [63] investigated the efficacy of phage treatment on nasal colonization of LA-MRSA in pigs. After demonstrating that MRSA could be inhibited by bacteriophages in vitro, an in vivo trial was conducted. Sixteen MRSA-negative piglets were intranasal colonized with MRSA. A few days later, a bacteriophage gel was applied to the nostrils of eight piglets for five consecutive days, while the remaining eight piglets served as a control group. The presence of MRSA and bacteriophages was monitored throughout the study. The results showed no statistically significant reduction in MRSA colonization in the phage-treated group compared to the control group.

In their study, Honegger et al. [64] evaluated the effectiveness of phage treatment in reducing MRSA prevalence in pigs. The trial was conducted in three separate phases. During the first phase, MRSA-positive sows were treated with a bacteriophage cocktail applied to their skin, mouth, nose, and vagina prior to giving birth. After birth, sows and their piglets received a daily dose of phages in their feed, and both sows and piglets were sprayed with the phage solution twice a week. As the treatment showed no significant effect on MRSA decolonization, the phage concentration was increased for the piglets in the second and third phases and administered through their drinking water. Additionally, the barn was nebulized with phages three times a day.

At the end of the second phase, all piglets were MRSA-positive. In contrast, none of the piglets in the third phase tested positive for MRSA. However, the authors cautioned against overinterpreting these results, as only one of 40 piglets was MRSA-positive at the beginning of the third phase, compared to 11 of 44 piglets in the second phase. Overall, the authors concluded that phage treatment was not effective in decolonizing pigs from MRSA.

### Washing of sows

Washing sows before moving them into the farrowing pen is often cited as an important biosecurity measure. This practice is primarily aimed at removing dirt and germs from the sows, thereby minimizing transmission to suckling piglets [65]. Studies by Pletinckx et al. [66] and by Verhegghe et al. [67] investigated the effectiveness of sow washing in reducing MRSA prevalence in pig farms and, consequently, the transmission to their piglets.

Pletinckx et al. [66] divided the sows into a test and a control group to assess the impact of washing and disinfection on MRSA prevalence. In addition to sampling the sows, samples were collected from the environment and piglets at different time points. The sows in the test group were first washed with a shampoo containing dodecyl dimethylamine oxide and then rinsed. Their skin was subsequently disinfected with a chlorhexidine and isopropanol solution. After washing, the sows were moved to the farrowing unit, which had been subject to C&D immediately beforehand. Process began with the removal of organic material, followed by cleaning using sodium capryliminoproprionate. High-pressure cleaning was then performed, and the area was left to dry for five days. Finally, disinfection was carried out using a combination of alkyldimethylbenzylammonium chloride, isopropanol, glutaraldehyde, and didecyldimethylammonium chloride. In the farrowing unit, the sows continued to receive daily disinfection treatments for an additional five days.

Piglets were born in the farrowing unit, and samples were taken from the sows and the environment immediately before and after sow washing. Further samples were collected from the piglets, sows, and environment on day 5 and before weaning (21–28 days). Additional samples from the piglets and their environment were taken on days 38 and 60 in the rearing unit.

The authors observed an initial decrease in MRSA prevalence in the test group, dropping from 64% before washing to 4% after treatment. In the control group, which did not undergo washing and disinfection, no significant reduction was observed, with MRSA prevalence remaining stable (70–72%). After the final disinfection (day 5), the MRSA prevalence was significantly lower in the test group (29%) compared to the control group (95%). However, by weaning (day 21–28), the difference between the two groups was no longer significant.

Interestingly, the piglets from the test group initially had a lower MRSA prevalence (58%) compared to those from the control group (84%). However, shortly before weaning, this difference was no longer significant. Overall, the study concluded that washing and disinfection had a temporary but significant effect on MRSA reduction, although no long-term impact was detected.

Verhegghe et al. [67] conducted a study on four farrow-to-finish pig farms to investigate the effects of sow washing on the presence of LA-MRSA. Twelve sows were sampled per farm, and the farms were divided into two groups based on different washing protocols. On Farms A and B, sows were washed in the gestation unit before being moved to the farrowing unit. On Farms C and D, sows were moved to a previously disinfected farrowing unit and washed there.

All sows were initially rinsed with water. Farms A and B manually applied the cleaning agents with a contact time of 5 min, while farms C and D used high-pressure applications with a contact time of 15 min. The cleaning agents varied between farms: farms A and C used a combination of glutaraldehyde and methylisothiazolinone, Farm B used sodium hydroxide and sodium metasilicate, and Farm D applied a mixture of soap and coconut oil. After the contact period, the sows were rinsed again with water.

Samples were collected from the sows’ skin and nostrils immediately before and after washing. Results varied between farms, but overall, the authors found that sow washing had no significant effect on reducing the MRSA prevalence. However, washing seemed to have a greater impact on skin samples than on nasal samples. On Farm A, most sows were negative both before and after washing, while on farm B, six sows tested positive for MRSA after washing despite being negative beforehand. Farms B and C had a high number of LA-MRSA-positive sows, and most of them remained positive after washing.

## Discussion

This narrative review aimed to identify and evaluate intervention strategies to reduce and prevent the occurrence of MRSA in pig farming. While good hygiene and management practices are fundamental to animal husbandry, this review focuses specifically on assessing targeted measures [68]. Of 2.605 publications retrieved, 21 studies were selected for inclusion. The transmission of MRSA among pigs, as well as its zoonotic potential, poses a significant global public health concern. Addressing this issue requires the implementation of targeted intervention measures. Given the multiple transmission pathways between pigs, and between pigs and humans [69], it is essential to consider a variety of intervention approaches. Furthermore, understanding the key risk factors for introduction, emissions, and spread of MRSA is crucial for effectively categorizing and prioritizing intervention strategies.

One commonly cited risk factor for the spread of MRSA in pig farms is the use of antibiotics. The studies included in this review yielded varying results. For instance, Dorado-García et al. [39] reported a reduction in MRSA prevalence following reduced antibiotic use, whereas Lopes et al. [40], and Dierikx et al. [35] did not observe such a reduction. Numerous studies have demonstrated that the use of antibiotics, particularly group treatments, is a risk factor for MRSA carriage. Conversely, restricted use of antibiotics has been associated with lower MRSA prevalence in pig herds [70–72]. It is also assumed that reduced antibiotic use leads to decreased MRSA transmission rates [73]. This hypothesis is supported by simulation studies conducted by Sørensen et al. [74] and Schulz et al. (a) and (b) [75, 76], who concluded that reducing antibiotic use decreases MRSA prevalence but does not result in its complete elimination. These findings are consistent with those of Dorado-García et al. [39].

The differing results in the study by Dierikx et al. [35] could be attributed to the sampling location, as this study collected samples in abattoirs unlike the other studies [39, 40]. It is possible that cross-contamination occurred during transportation or while the pigs were in the slaughterhouse, which could have influenced the results [35, 38]. A further factor to consider is that Lopes et al. [40] focused specifically on the reduced use of colistin and amoxicillin for prophylactic purposes in feed in the investigated farms, rather than on overall antibiotic use. The continued use of tetracyclines, along with the high resistance rates of MRSA to tetracyclines, remained significant. Additionally, tetracyclines have a long persistence in the environment, which could explain why the expected reduction in MRSA prevalence was not observed [77]. The prophylactic use of antibiotics was severely restricted in Regulation (EU) 2019/6 of the European Parliament and of the Council of 11 December 2018 on veterinary medicinal products and repealing Directive 2001/82/EC which had to be implemented in 2022 only. This means that in the future antibiotics may only be administered in exceptional cases, and only to a limited number of animals if the risk of infection is very high and the consequences are serious.

C&D are critical biosecurity measures in livestock management, particularly in pig farming. Many of the studies included in this review focused on C&D as a strategy to prevent the spread of MRSA within farms. Despite differences in cleaning processes, study designs, and specific objectives, these studies consistently demonstrated that C&D is an effective method for reducing bacterial loads in pig holdings. However, there were variations in the cleaning processes, including the types of cleaning agents and disinfectants used. In recent years, resistance to various biocides has increased [78, 79], with quaternary ammonium compounds being frequently implicated. This resistance may partially explain the reduced effectiveness of C&D observed in some studies. The extent to which biocide resistance impacted the effectiveness of C&D in individual studies could not be fully assessed.

Luyckx et al. (a) [49] investigated an alternative cleaning method using *Bacillus* spp. spores as a CE approach due to concerns about resistance. However, their method proved less effective than traditional C&D, likely because the bacterial load administered to pigs and their environment via spray application was insufficient. Another factor influencing the effectiveness of C&D is the interior design of pig barns. For example, higher bacterial counts were found in areas such as drinking nipples, while feed troughs showed lower contamination [46, 49, 50]. In addition, it is important to thoroughly clean areas outside the pig housing, as these areas remained positive for LA-MRSA after C&D in contrast to samples taken at the animals‘ height [42]. These findings should be considered when developing C&D protocols.

Despite successful C&D, many studies reported a resurgence of MRSA prevalence after pigs were restocked [42, 43, 49]. Several factors may explain this aspect. One obvious and very relevant factor is the purchase of MRSA-positive pigs, which significantly increased MRSA prevalence in both the pigs and their environment, as pigs are considered the primary carriers of the pathogen [70, 73, 80]. Several studies demonstrated that introducing MRSA positive pigs into a barn might lead to a rapid increase of MRSA prevalence in pigs [42, 43, 49]. Another potential factor could be human-related transmission. Workers may inadvertently introduce MRSA into cleaned areas by wearing contaminated clothing or failing to decolonize themselves after exposure to MRSA-positive environments [42]. Studies have shown that people who work with pigs have significantly higher MRSA colonization rates, making this a plausible route of reintroduction [13, 14].

A study by Schollenbruch et al. [27] offered an interesting perspective. They found that while MRSA prevalence initially increased after successful C&D on farms with straw bedding, it decreased after a few weeks in both pigs and the environment. The authors attributed this reduction to the competitive effect of bacteria in the straw. Straw bedding is commonly used in organic farming systems. Other studies support the findings of Schollenbruch et al., suggesting that straw bedding may contribute to lower MRSA and AMR rates on pig farms [18, 27, 81–83]. However, these studies also noted that straw bedding cannot be viewed isolated, as other aspects, such as smaller herd size, improved ventilation, and reduced antibiotic use, also play a role in these farming systems.

An alternative approach to preventing MRSA spread within farms is the eradication of MRSA-positive pigs, as examined in two Norwegian studies. Norway, unlike countries with higher MRSA prevalence such as Germany and Denmark, has implemented an MRSA eradication program and maintained low MRSA prevalence at national level. The program includes identifying positive herds through continuous monitoring and contact tracing. For herds identified as positive, the protocol involves eradicating infected animals and conducting comprehensive C&D of the barns. Only animals confirmed as MRSA-negative are reintroduced, following a successful efficacy test of the C&D process. The difference in baseline prevalence between Norway and for example Germany significantly impacts the feasibility and effectiveness of such interventions. Both studies demonstrated that eradication can successfully control MRSA on previously positive farms [52, 53], suggesting that eradication is both effective and feasible in a low-prevalence setting such as Norway. However, in countries with high prevalence, such as Germany, culling all MRSA-positive animals may not be justifiable due to increased likelihood of MRSA reintroduction through the purchase of infected pigs, ethical concerns and substantial costs [43].

Various methods of air cleaning are grouped under the term “air filtration” in the results, as they all address the transmission of MRSA through the air. A distinction must be made between exhaust air filtration and air filtration within barns. Exhaust air filtration is used to prevent the discharge of MRSA-contaminated air, while air filtration inside the stables is primarily aimed at reducing the spread of MRSA within the facility [54–56, 58]. Exhaust air filtration systems are commercially used in pig farms and are mandatory e.g., in Germany under specific conditions [84]. These systems aim to reduce emissions such as ammonia, odors, and dust from exhaust air [84, 85]. Additionally, some studies indicate that these systems can also reduce the number of microorganisms, including MRSA [86, 87].

Air filtration systems can be categorized into biofilter, exhaust air, chemical, and multistage systems, each with its own advantages and disadvantages in emission reduction [84, 85]. However, none of the studies reviewed investigated the MRSA removal efficacy of chemical systems, which represents a research gap. Air filtration using biofilters, exhaust air, and multistage systems has proven effective in pig farms [54–56]. Nonetheless, further research is necessary due to the influence of various factors, as demonstrated by the inconsistent results of Clauss et al. [54]. For example, only a small number of farms were examined, and the investigations did not account for changes in climatic conditions, such as relative humidity and temperature, making further research essential [88]. Moreover, pig sheds differ in construction, feeding technology, underfloor extraction systems, size, and other factors that influence the efficiency of air filtration systems, highlighting the need for more comprehensive studies [54, 84, 85].

Other methods explored for reducing MRSA on pig farms include bacteriophage cocktails and sow washing [62–64, 66, 67]. Although bacteriophage application is gaining popularity in human medicine, and successful applications have been documented in veterinary medicine [59, 89, 90], none of the studies reviewed showed a satisfactory reduction of MRSA using this method [62–64]. At its current stage, phage therapy does not appear to be a viable solution for eradicating MRSA from pig farms. For more effective use, further optimization of bacteriophage cocktails, increased phage concentration, or targeting pigs with higher MRSA concentrations may be necessary.

Sow washing prior to moving them into the farrowing pen was also investigated as a method for reducing MRSA on pig farms. The two studies reviewed yielded differing results. Pletinckx et al. [66] achieved at least a temporary reduction in MRSA prevalence, whereas Verhegghe et al. [67] observed no reduction. This discrepancy may be due to differences in washing protocols, such as the absence of sow skin disinfection following washing in the study by Verhegghe et al. [67]. Generally, questions remain regarding the practicality of sow washing due to associated costs and labor demands.

In addition to animal-to-animal transmission, humans are recognized as a potential source of MRSA in livestock environments [12–15]. Studies showed that individuals who work closely with animals have higher rates of MRSA colonization than the general population, emphasizing the zoonotic nature of LA-MRSA and the bidirectional risk of transmission [13, 39]. The role of humans as carriers can complicate MRSA control efforts. Even with stringent biosecurity and disinfection protocols, workers may inadvertently reintroduce MRSA to sanitized environments or spread it between farms through contaminated clothing or equipment. For example, Kobusch et al. (42) discussed that despite thorough decontamination measures on a model pig farm, MRSA reappeared within weeks. This was potentially influenced by human-related factors, including cross-contamination from workers or other external sources. Such findings highlight the importance of consistent barrier measures, such as changing boots and clothing, handwashing, and wearing respiratory masks, to minimize LA-MRSA carryover during transitional phases of decontamination [42].

Addressing this risk involves implementing targeted hygiene practices, such as mandatory protective clothing, hand sanitization, and changing facilities for workers entering and leaving MRSA-positive areas [43]. Regular screening of personnel and, where feasible, decolonization strategies could further reduce the likelihood of MRSA transmission from humans to animals [22]. Additionally, training farm workers on MRSA risks and proper hygiene practices can reinforce biosecurity efforts, making human-related transmission a manageable factor in comprehensive MRSA control strategies.

The complexity of MRSA transmission dynamics in livestock environments, particularly in pig farming, suggests that single interventions may be insufficient for sustainable control. Studies show that combining multiple intervention strategies, such as biosecurity measures, antimicrobial use reduction, and environmental disinfection, can be more effective than any single measure alone [14, 22, 39]. This aligns with findings from simulation studies by Schulz et al. (a) and (b) [75, 76] which also indicated that a combination of multiple measures most effectively reduces MRSA prevalence in pig herds. Such findings underscore the need for a holistic approach tailored to each farm’s specific conditions, especially in regions with high MRSA prevalence.

## Conclusion

The spread of MRSA in pig herds poses major challenges for both agriculture and policy-making. Various intervention strategies are therefore being investigated to reduce MRSA prevalence in pig farms. This comprehensive study identified multiple measures, including the reduction of antimicrobial use, C&D, eradication, air filtration, bacteriophage application, and sow washing, each demonstrating varying degrees of effectiveness. The analysis of these strategies revealed that no single measure alone ensures sustainable MRSA reduction. Instead, a combination of targeted interventions such as enhanced biosecurity, reduced antimicrobial use, environmental decontamination, and, where feasible, eradication programs, offers the greatest potential for effective MRSA control. A holistic approach is therefore essential to reducing MRSA prevalence in pig farming and supporting the global One Health initiative.

## Data Availability

No datasets were generated or analyzed during the current study.

## Abbreviations

AMR: Antimicrobial resistance
CA-MRSA: Community-associated Methicillin-resistant Staphylococcus aureus
C&D: Cleaning and disinfection
CE: Competitive exclusion
ECAS: Electrochemically activated solution
EFSA: European Food Safety Authority
EU: European Union
HA-MRSA: Hospital-associated Methicillin-resistant Staphylococcus aureus
LA-MRSA: Livestock-associated Methicillin-resistant Staphylococcus aureus
MRSA: Methicillin-resistant Staphylococcus aureus
WHO: World Health Organization
